# Determinants of Tuberculosis Treatment Outcomes in Patients with TB/HIV Co-Infection During Tuberculosis Treatment at Selected Level One Hospitals in Lusaka, Zambia

**DOI:** 10.3390/antibiotics14070664

**Published:** 2025-06-30

**Authors:** Theresa Musa Hassab, Audrey Hamachila, Aubrey Chichonyi Kalungia, Norman Nyazema, Moses Mukosha, Chikafuna Banda, Derick Munkombwe

**Affiliations:** 1 Department of Pharmacy, University of Zambia, Lusaka 10101, Zambia; hachuuludm@yahoo.co.uk (T.M.H.); audrey.hamachila@unza.zm (A.H.); ckalungia@unza.zm (A.C.K.); moses.mukosha@unza.zm (M.M.); 2 Department of Pharmacy, University of Limpopo, Mankgweng 0727, South Africa; nnyazema@zimpharm.co.zw; 3 Department of Biomedical Sciences, Copperbelt University, Ndola 10101, Zambia; chikafunaj.banda@cbu.ac.zm

**Keywords:** TB/HIV co-infection, viral load, sputum smear conversion, comorbidity, treatment outcomes, Zambia

## Abstract

**Background/Objectives:** Tuberculosis (TB) and HIV co-infection pose significant challenges in resource-limited settings, contributing to multi-drug-resistant TB when treatment fails. This study aimed to identify determinants of TB treatment outcomes among HIV/TB co-infected patients in Lusaka, Zambia. **Methods:** A retrospective cohort study was conducted at Chilenje, Chipata, and Chawama level one hospitals, using systematic sampling to select 586 patient files. Data were analyzed with SPSS version 23, employing descriptive statistics, chi-square tests, and hierarchical logistic regression. **Results:** Among the study population (*n* = 586), consisting predominantly of working-age adults (25–44 years: 61.6%) and males (56.5%), treatment success was 81.3%, with a 12.5% mortality rate across treatment phases. Baseline smear-negative TB, viral load (100,000–199,999 copies/mL), diabetes without hypertension, and negative smear at follow-up independently predicted treatment outcomes. Higher treatment failure odds were linked to smear-negative TB, high viral load, and hypertension–diabetes comorbidity, while CD4 count and HIV treatment status showed no independent effects. **Conclusions:** These findings underscore the influence of viral load, TB type, comorbidities, and sputum conversion on treatment success, emphasizing the need for targeted monitoring and integrated care, particularly in the continuation phase, to enhance outcomes in this vulnerable population.

## 1. Introduction

The two infectious diseases, Tuberculosis (TB) and Human Immunodeficiency Virus (HIV), are major health challenges worldwide and also the leading cause of public health burden in resource-limited countries [1]. TB has been cited to be the second leading cause of deaths among curable infectious diseases; about one-third of populations worldwide are infected. According to a World Health Organization (WHO) report, the incidence of new infection is at a rate of one per second on a global scale [2]. It is well known that TB is one of the major critical health problems and the rise in the cases of MDR-TB has further affected the control of TB worldwide [3]. Unsuccessful treatment of drug-susceptible TB in TB/HIV co-infected individuals has been cited to be among the major causes of MDR-TB and a threat to its control [4].

The HIV pandemic presents a massive challenge to the control of TB at all levels [5]. The synergy between TB and HIV is strong; in high HIV-prevalent populations, TB is one of the world’s most common causes of death and is one of the most common chronic infectious conditions causing mortality and severe outcomes, particularly in people living with HIV/AIDS [6]. It is also among the leading causes of deaths, especially for populations in sub-Saharan Africa [7]. HIV promotes progression of latent or recent infections of Mycobacterium Tuberculosis to active diseases and also increases the rate of occurrence of TB. From infectious diseases’ statistics, TB and HIV are the leading causes of death globally. Despite the many efforts that have been made to control and prevent the dual occurrences of the diseases, TB is still the leading cause of mortality and morbidity in people living with HIV [8].

Hamad, Kwami, and Ahmed [3] stated that HIV/TB co-infection management is a complicated endeavor, with huge treatment failure and resistance to antimicrobials. They further stated that, overall, one in approximately seven of TB/HIV co-infected adults had experienced treatment failure with an incidence rate of 4.5 per 100 PYs.

In Zambia, according to the Zambian national strategic plan for TB and Leprosy prevention, care, and control of 2022–2026, the HIV/TB burden continues to cause an enormous health challenge to the Zambian people as well as to the Zambian healthcare system. In 2020, the incidence of TB cases was estimated to be 59,000, equivalent to a rate of 319 per 100,000 populations. Furthermore, HIV/TB co-infection presents an economic challenge because the government spends colossal sums of money to procure TB drugs and other equipment to help combat the illness. The survey reported a TB prevalence of 319/100,000 and that the risk of acquiring TB was five times higher in HIV-positive than in HIV-negative individuals [9]. As of the year 2015, deaths due to TB have been estimated to be at 22.0 per 100,000 population [10].

Several strategies and policies have been put in place globally in order to end the TB disease burden. For instance, the WHO declared TB as a global health emergency in 1993, and in 1994–1995, it launched a five-element DOTS strategy that was adopted by most high-TB burden countries, including Zambia [11]. In 2014 and 2015, the member states of the WHO and the UN committed to ending the TB epidemic by endorsing the WHO’s End TB strategy, whose overall goal was to end the TB epidemic globally by addressing major old and new constraints to TB control while taking advantage of new opportunities. The Zambian Ministry of Health (MOH), during its commemoration of World TB Day in 2019, launched the guidelines for the management of latent TB infection in order to strengthen TB treatment at the primary healthcare level [12].

Despite the efforts made recently in the control of TB, the mortality among people living with TB/HIV is still high [13]. Furthermore, despite the existence of ART, mortality among people who are co-infected with TB and HIV has not decreased. Although co-infection with TB and HIV is bidirectional and a dual public health burden worldwide, TB/HIV co-infected patients have not received enough attention [14]. Zambia has seen a rapid rise in TB cases [15], and TB continues to be a public burden in Zambia. Unfortunately, the predictors of unsuccessful TB treatment outcomes as well as risk factors associated with unsuccessful treatment outcomes during the intensive phase of TB treatment have not been fully described in Zambia [16]. Thus, the aim of this study was to assess predictors of Tuberculosis treatment failure and mortality in patients with HIV/TB co-infection during TB treatment at selected level one hospitals in Zambia.

## 2. Results

The data analysis proceeded through three main phases: descriptive analyses, bivariate analyses, and multivariable regression.

First, descriptive statistics were calculated to summarize the demographic, clinical, and treatment-related characteristics of the study population. Second, bivariate analyses were performed to explore associations between potential predictors and the primary outcome. Finally, a hierarchical logistic regression analysis was employed to assess the independent effects of key predictors on treatment outcomes.

### 2.1. Demographic and Clinical Characteristics of the Study Sample

This study included 586 participants, whose demographic and baseline clinical characteristics are summarized in Table 1.

The majority of patients (50.9%, *n* = 298) were from the Chilenje hospital, followed by the Chipata (27.8%, *n* = 163), and the least number were from the Chawama hospital (21.3%, *n* = 125). The unbalanced representation of study participants from each hospital may reflect differences in patient populations or referral patterns.

The study population exhibited a broad age range, with the largest proportion of participants falling within the 25 to 44 age group (61.6%, *n* = 361). A substantial proportion was also in the 45 to 64 age range (23.7%, *n* = 139), while 9.7% of participants were between 18 and 24 (*n* = 57). A relatively small number of participants were under 18 years old (0.2%, *n* = 1) or 65 years and older (4.4%, *n* = 26). Finally, the study population comprised a higher proportion of males (56.5%, *n* = 331) compared to females (43.3%, *n* = 254).

The distribution of baseline BMI shows that nearly half of the participants were within the normal weight range (BMI 18.5 to 24.9) at 50% (*n* = 289). A substantial proportion were underweight (26.1%, *n* = 151), while 16.4% (*n* = 95) were overweight and a small proportion were obese (7.4%, *n* = 43). The baseline viral load distribution showed a varied range, with a large proportion of participants having relatively high viral loads (10,000 to 99,999 copies/mL) at 43.5% (*n* = 255). Approximately 14.2% (*n* = 83) had the highest viral loads (200,000 and above), and 22% (*n* = 129) had viral loads between 100,000 and 199,999 copies/mL. Meanwhile, 16% (*n* = 94) had viral loads between 1000 and 9999 copies/mL, and 4.3% (*n* = 25) had suppressed viral loads (under 1000 copies/mL). The baseline CD4 cell count showed that over 70% of the participants had low CD4 cell counts at enrolment. A majority of participants had CD4 counts between 200 and 349 (40.9%, *n* = 238), and another 31.8% (*n* = 185) had severely depressed CD4 counts under 200. Only 17.4% (*n* = 101) had CD4 counts between 350 and 500, and 10% (*n* = 58) had CD4 counts over 500. The distribution of HIV status at enrolment was roughly balanced, with 43.4% (*n* = 252) being newly diagnosed with HIV at the time of TB diagnosis, while 56.6% (*n* = 328) were already on ART. The majority of participants had smear-negative TB (78.8%, *n* = 461), compared to smear-positive TB (21.2%, *n* = 124). This may be due to more effective screening for sputum-positive TB or a combination of factors, resulting in more participants with sputum-negative or extra-pulmonary TB. The majority of participants were new TB cases (66.5%, *n* = 389), while a substantial proportion had a history of previous TB treatment, with 33.5% (*n* = 196) of participants being retreatment cases. The high proportion of retreatment cases is an important concern, indicating the need to address treatment adherence and efficacy of TB therapy. The majority of study participants had no other recorded comorbidities (83.4%, *n* = 487), while 16.4% (*n* = 96) reported at least one other comorbidity. Of those reporting comorbidities, 87.2% (*n* = 509) had hypertension/diabetes, while 8.7% (*n* = 51) had hypertension and 4.1% (*n* = 24) had diabetes.

At the end of the intensive phase, smear conversion status was assessed. Among participants who were initially smear-positive and had follow-up data available for smear conversion (*n* = 585 total with data), the majority, 90.1% (*n* = 527), achieved smear conversion, meaning their sputum smear result became negative. A small percentage, 0.3% (*n* = 2), remained smear-positive at this time point. Data on smear conversion were not available for 9.6% of participants (*n* = 56).

### 2.2. Mortality Rates Between the Intensive and Continuous Phases

The treatment outcomes of the study participants are summarized in Table 2.

At the end of the intensive phase of TB treatment, the vast majority of participants were alive (93.1%, *n* = 541). A total of 40 participants died during the intensive phase of treatment, representing 6.9% of the study sample. This suggests a relatively low mortality rate during this phase of treatment.

At the end of the standard 6-month TB treatment period, 11.8% (*n* = 69) of participants were classified as cured, and 69.5% (*n* = 405) had completed treatment. However, 6.2% (*n* = 36) were lost to follow-up, and 12.5% (*n* = 73) had died by the end of treatment. These results suggest a considerable treatment success rate; however, it also highlights significant challenges, as patients are dying during treatment.

### 2.3. Sputum Conversion Rates During Treatment

**Figure 1:**
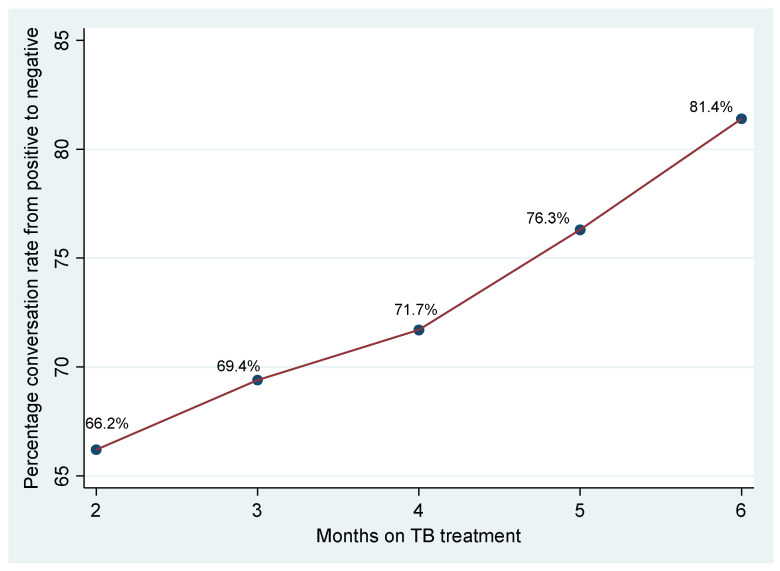
Sputum conversion rate from positive to negative over 6 months.

Sputum conversion, the transition from a positive to a negative sputum smear, is a crucial indicator of treatment response in pulmonary Tuberculosis (TB). For this study, the sputum conversion rates were measured at months 2, 3, 4, 5, and 6, as shown in Figure 1.

The figure illustrates the percentage of patients who achieved sputum conversion from a positive to a negative smear at various time points during the treatment period. At the end of the intensive phase, or month 2 of treatment, 66.2% of patients had achieved sputum conversion, indicating that approximately one-third of patients were still sputum-positive at this early point in treatment. The conversion rate showed a gradual increase in the subsequent months. At month 3, 69.4% of patients had achieved sputum conversion, indicating a modest increase from month 2. At month 4, 71.7% had achieved conversion, showing continued progress in treatment response. The rate of sputum conversion increased to 76.3% at 5 months, and the rate of conversion steepened in this period. By the end of the treatment period at six months, 81.4% of patients had converted from positive to negative smears, demonstrating that most patients achieved sputum conversion by this point.

### 2.4. Chi-Square Bivariate Analysis

To assess the relationships between various patient characteristics and treatment outcomes at the end of 6 months, chi-square tests were performed. The results are summarized in Table 3.

Using Chi-Square analysis, statistically significant associations were observed for baseline viral load (χ^2^ = 39.080, df = 12, *p* < 0.001), baseline CD4 (χ^2^ = 19.807, df = 9, *p* = 0.019), category of HIV (χ^2^ = 14.371, df = 3, *p* = 0.002), type of TB (χ^2^ = 291.250, df = 3, *p* < 0.001), comorbidity type (χ^2^ = 14.222, df = 6, *p* = 0.027), and smear conversion at follow-up (χ^2^ = 64.824, df = 6, *p* < 0.001). In contrast, no statistically significant associations were found for age (*p* = 0.176), sex (*p* = 0.641), baseline BMI (*p* = 0.343), category of TB (*p* = 0.114), other comorbidities (*p* = 0.577), and intensive phase duration (*p* = 0.100). The lack of significant association with age, sex, baseline BMI, category of TB, other comorbidities, and intensive phase duration suggests they may not be critical determinants of treatment outcome in this study. These variables were not considered for multivariate analysis

### 2.5. Hierarchical Logistic Regression Analysis

To assess the independent and cumulative effects of various patient characteristics on TB treatment outcomes, a hierarchical logistic regression analysis was conducted. The results are presented in Table 4.

Table 5 below shows a summary of the logistic regression analysis that was carried out to determine the antecedents of treatment outcomes in patients with TB/HIV co-infection.

This discussion focuses on the final iteration of this model, Model 6, which incorporated baseline viral load, baseline CD4 count, category of HIV, type of TB, comorbidity type, and smear conversion status at follow-up.

The overall statistical adequacy of Model 6 was confirmed. The Omnibus Test of Model Coefficients revealed that the collective set of predictors included significantly enhanced the prediction of TB treatment outcomes when compared to a null model devoid of predictors, as evidenced by a chi-square value of 35.24 with a *p*-value of less than 0.001. The explanatory power of the model, indicated by the Nagelkerke R^2^ value, was 0.200, suggesting that approximately 20.0% of the variance in treatment outcomes could be attributed to the included predictors; the Cox and Snell R^2^ was 0.123. Moreover, the Hosmer and Lemeshow test indicated good model calibration, with a chi-square of 5.939 and a *p*-value of 0.654, signifying no statistically significant disparity between the observed and model-predicted frequencies of treatment outcomes. The −2 Log likelihood for this final model was 473.09, further supporting its fit.

Examining the individual predictors within Model 6, several factors emerged as significant influences on TB treatment outcomes. Regarding baseline viral load, with a viral load under 1000 copies/mL serving as the reference, patients presenting with a baseline viral load between 100,000 to 199,999 copies/mL demonstrated significantly diminished odds of achieving a successful TB treatment outcome (OR = 0.319, *p* = 0.016). This indicates their odds of success were approximately 68.1% lower than the reference group. Other viral load categories did not show a statistically significant independent effect in this final adjusted model.

Conversely, baseline CD4 count, with a count under 200 cells/µL as the reference, did not emerge as an independent predictor. None of its categories (200–349, 350–500, or over 500 cells/µL) were significantly associated with treatment outcome after accounting for other variables. Similarly, the category of HIV, likely comparing patients already on ART to those newly diagnosed (with newly diagnosed as the reference), was not found to be significantly associated with treatment outcomes in the final model (OR = 1.117, *p* = 0.708).

The type of TB at diagnosis was a significant predictor. Patients diagnosed with smear-negative TB (with smear-positive TB as the reference) exhibited significantly lower odds of a successful treatment outcome (OR = 0.277, *p* = 0.001), representing an approximate 72.3% reduction of the odds of success compared to their smear-positive counterparts.

Comorbidity type also played a role. With hypertension alone as the reference category, patients with diabetes mellitus alone had significantly lower odds of a successful TB treatment outcome (OR = 0.300, *p* = 0.039), a 70% reduction of the odds. The presence of both hypertension and diabetes, however, did not show a statistically significant difference in outcome compared to hypertension alone (OR = 2.058, *p* = 0.169).

Smear conversion status at follow-up was a particularly strong determinant of treatment outcome, with a positive smear at follow-up as the reference. Patients who achieved negative smear conversion displayed exceptionally high odds of a successful treatment outcome (OR = 6.75 × 10^8^, *p* = 1.00). This remarkably high odds ratio, coupled with a *p*-value of 1.00, suggests quasi-complete separation in the data, indicating a very strong positive predictive relationship for success. In contrast, patients whose smear conversion status was unknown, missing, or not performed had significantly lower odds of a successful outcome (OR = 0.137, *p* = 0.001) compared to those who remained smear-positive, translating to an 86.3% decrease of their odds of success.

## 3. Discussion

### 3.1. Treatment Outcome Ratios

The study found that during the period 2020–2022 at Chilenje, Chawama, and Chipata level one hospitals in Lusaka, Zambia, 18.7% of patients had unsuccessful TB treatment outcomes (defined as death or lost to follow-up), with a corresponding treatment success rate of 81.3%. The finding of this study showed a substantial improvement (25.1%) than what was reported by the local study [18], which found a 43% unsuccessful treatment outcome. This improvement could be due to increased TB awareness, early TB detection and treatment commencement, short distances from patients’ homes and level one hospitals, a well-enhanced DOTS program, TB adherence supporters, and HIV/TB-integrated patient care. The results were also slightly (3.1%) lower than the African systemic review, which recorded a 21% unsuccessful TB treatment outcome [19]. Furthermore, the successful TB treatment outcome in this study was slightly lower than the WHO’s set target for Millennium Development Goals (MDGs) at about 85% and far lower than the End TB strategy’s milestone success rate set for the year 2025 by more than 90% [2]. The fact that these level one hospitals are situated in the vicinity of most patients’ communities and homes, fear of discrimination and stigma within the community might largely be the contributing factor to unsuccessful TB treatment.

### 3.2. Sputum Conversion Rates

The observed sputum conversion rate of 81.4% at six months falls below the 90–95% typically reported even among TB/HIV co-infected cohorts. For instance, in a cohort of HIV-positive patients in Ethiopia, 94% achieved conversion by month 5 of therapy (88% at month 2 and 94% at month 5) [20], and peripheral laboratory data have documented a 94.9% conversion rate at eight weeks in Category I (new) cases [21]. Several hypotheses may explain our lower rate:

Higher baseline bacillary burden: Patients presenting with heavy smear grading (e.g., 3+) require more prolonged therapy to achieve negativity. Studies have shown that a baseline smear grade > 2+ is associated with delayed conversion (adjusted odds ratio ≈ 3.8) [22]. Although multidrug-resistant cases were excluded, primary mono- or poly-resistance, unmeasured in this cohort, could similarly slow clearance.

Immunosuppression and CD4^+^ count: Advanced HIV disease impairs granuloma formation and bacillary killing. In a South African cohort, lower CD4^+^ counts (<200 cells/µL) were independently linked to a 30% reduction of two-month conversion rates [23]. Our dataset did not incorporate baseline CD4^+^ strata in conversion analyses, but given that 15% of the participants initiated ART within four weeks of TB therapy, many may have remained profoundly immunosuppressed during the intensive phase.

Nutritional status and pharmacokinetics: Underweight patients (BMI < 18.5 kg/m^2^) exhibit altered drug absorption and distribution, delaying sputum conversion (AOR ≈ 0.54 for conversion at one month) [22]. Malnutrition is highly prevalent in Lusaka TB clinics and may have contributed to slower smear negativity.

Adherence and programmatic factors: Interruptions in therapy, even brief, can lead to persistent positivity. Digital pillbox monitoring in urban Zambia found that 20% of missed doses during the intensive phase doubled the hazard of non-conversion at two months [24]. Although our program employs directly observed therapy (DOT), resource constraints and transportation barriers could undermine adherence.

To rigorously elucidate these determinants, future studies should consider a multivariable logistic regression of end-of-treatment conversion (dependent variable) against: baseline smear grade, CD4^+^ count category, BMI, timing of ART initiation, and adherence metrics (independent variables). Interaction terms between CD4^+^ strata and smear grade may reveal synergistic effects on conversion delays.

### 3.3. Mortality Rates During Treatment

Our analysis showed that 6.9% of participants died by the end of the intensive phase and 12.5% by treatment completion. Although this overall mortality is lower than the 16–24% [25] typically reported among TB/HIV cohorts, several factors, most notably the age structure of our study population, likely account for this difference.

The predominance of younger adults (25–44 years) in our sample confers a survival advantage. Younger patients generally have fewer chronic comorbidities and stronger immune function, enabling better tolerance of both anti-TB and antiretroviral therapies.

A meta-analysis in Ethiopia [26] reported a pooled TB/HIV mortality of 16.2% (95% CI: 13.0–19.2) and found age > 45 years to be an independent risk factor for death (adjusted hazard ratio = 2.05; 95% CI: 1.03–4.12). In contrast, only 28.3% of our cohort were aged ≥ 45 years, further explaining our lower rate.

Additionally, since the introduction of programmatic factors in Zambia, such as the “test-and-treat” policy in 2016, over 90% of TB/HIV patients initiated ART immediately upon HIV diagnosis, mitigating immunosuppression and reducing severe TB presentations [27].

Taken together, our relatively young cohort, high ART coverage with early initiation, and strengthened adherence mechanisms likely underly the 12.5% treatment completion mortality. Nonetheless, the bulk of deaths (6.9%) in the intensive phase highlights the need for intensified clinical monitoring, prophylactic cotrimoxazole, and nutritional support to further reduce early fatalities.

### 3.4. Factors Influencing Treatment Outcomes

The hierarchical logistic regression analysis identified several key independent predictors of TB treatment outcomes, providing insight into the factors that are most strongly associated with treatment failure among individuals with TB/HIV co-infection.

The results revealed that a baseline viral load between 100,000 to 199,999 copies/mL was associated with increased odds of treatment failure compared to those with the lowest viral load. Other levels of baseline viral load were not significantly associated with treatment outcome. This suggests that for, patients with high viral loads, additional interventions may be required and that further work is needed to determine how viral load can be used to predict poor treatment outcomes. The importance of viral suppression for TB treatment outcomes aligns with the findings of Geiger et al. [28], who showed that a lack of viral suppression at MDR-TB treatment initiation is associated with poor outcomes.

Individuals with smear-positive TB had statistically significant increased odds of treatment success compared to those with smear-negative TB (Exp(B) = 0.277, *p* < 0.001). These findings align with some previous studies, such as that by Kazemian et al. [29], who found that smear-positive TB is an independent predictor of favorable treatment outcomes. The results of our analysis highlight the increased risk associated with smear-negative TB and suggest the need for more tailored support and monitoring during the intensive treatment period for these patients, given the likelihood of worse treatment outcomes.

The presence of diabetes was associated with better treatment outcomes (Exp(B) = 0.300; *p* = 0.039) while having both hypertension and diabetes was associated with increased odds of treatment failure. Our study contrasts with some previous research that suggests that diabetes is an independent risk factor for developing unfavorable TB outcomes, as shown by Massavirov et al. [30] and Houck et al. [31], who found that diabetes and HIV were associated with an increased risk of mortality. The different results found in our study as compared to other work may be because many of the previous studies did not control confounders, and they may not have included smear conversion or viral load in their models. The reasons for the lower odds of treatment failure among those with diabetes and the higher odds of treatment failure when diabetes and hypertension are both present should be explored further in future research, with larger sample sizes and data on the duration of illness.

A negative smear at follow-up was associated with increased odds of treatment success as compared to those with a positive smear (Exp(B) = 0.137, *p* < 0.001). This finding underscores the importance of early treatment response. Consistent with other studies, this suggests that monitoring smear results at the end of the intensive phase is a useful way of predicting treatment success and for identifying those that may need more care. Kiplimo et al. [32], for example, demonstrated that delays in sputum conversion during treatment are associated with poor TB outcomes.

In contrast to the findings of some other studies [1,33], age, sex, and CD4 were not independent predictors of treatment outcomes, and having TB/HIV did not predict worse outcomes in this study, in contrast with Ruseesa et al. (2023) [34], who found that those not on ART were more likely to have unsuccessful treatment outcomes. This may suggest that the impact of these factors is mediated by the other variables included in the model, such as viral load.

The significant association between baseline viral load with treatment outcome supports the idea that effective ART initiation and monitoring are crucial for this population. High viral loads reflect poor viral suppression, which is an important target of public health intervention and must be addressed since it also affects TB outcomes. The increased odds of success among those with smear-positive TB may represent an advantage associated with this kind of disease, which may be easier to treat than sputum-negative TB. The decreased odds of success in those with diabetes and hypertension indicates the need for better diabetes management in patients with TB, a focus on comorbidities, and an area for further research. Smear conversion is an important predictor, as previously shown, and suggests that monitoring smear conversion early in treatment can be useful for predicting treatment success and identifying those who may need more care.

The findings from this study have policy and practice implications for the management of TB/HIV co-infection. The importance of baseline viral load and smear status highlights the need for more integrated TB and HIV care. This includes timely initiation of ART, adherence support, viral load monitoring, and ensuring patients receive an early smear test during the intensive phase of treatment. The association with both diabetes and the combination of diabetes and hypertension suggests the need for careful comorbidity screening, monitoring, and management for all patients receiving treatment for TB. Our findings highlight that patients with high viral loads and those with smear-positive TB need more monitoring. The early identification of these patients can allow healthcare providers to implement targeted interventions to better improve their treatment outcomes. Given that a large proportion of patients are lost to follow-up, more research is needed to identify and address the factors that contribute to this loss.

### 3.5. Research Recommendations

Based on the findings and limitations of this study, the following recommendations are proposed for policy, practice, and future research. Policy should emphasize the importance of integrating TB and HIV services to provide patients with comprehensive care. This should include ensuring that all HIV/TB patients receive ART, and that viral load monitoring is routinely performed. ART initiation should happen simultaneously with TB treatment initiation. Policies and guidelines should prioritize strategies to improve viral load suppression in TB/HIV co-infected patients. This includes adherence support, counseling, and the option for drug resistance testing when poor response is detected, to improve treatment outcomes and to reduce the risk of drug resistance. National guidelines should mandate routine smear microscopy at the end of the intensive phase of treatment, and these data should be used for patient care and entered into data systems for future analysis. There is a need to implement routine screening for metabolic comorbidities, and these must be appropriately addressed during TB treatment, given that both diabetes and hypertension were seen to have an impact on treatment outcomes. National programs should develop and implement interventions that improve patient retention during care and address the challenges that cause patients to discontinue treatment.

Healthcare providers should prioritize early viral load testing upon diagnosis of TB and repeat testing at shorter, regular intervals to assess ART efficacy. Interventions should focus on improving ART adherence in those patients with higher viral loads. Patients with smear-negative TB should receive more intensified monitoring, especially during the intensive phase of treatment, as this study showed that they are at a higher risk of treatment failure. This could include more frequent clinical visits or access to additional support services. Patients should be screened for diabetes and hypertension upon diagnosis of TB. The results of our study have shown that diabetes alone was associated with better outcomes, while the combination of diabetes and hypertension was associated with poor outcomes, highlighting the need to address both comorbidities with a targeted approach. Treatment programs should provide tailored support that addresses specific patient needs, including access to mental health services, social support, and nutritional counseling, as needed.

### 3.6. Limitations of the Study

The findings of this study were based on patients who commenced and completed treatment at the three study sites. The treatment outcome of trans-in and trans-out patients was not determined. Also, this study was a retrospective cohort study design that depended on data collected from the patient’s files and the smart care system. Hence, the findings of this study solely depended on available data that was captured in these files and we could not capture other factors such as the patient’s educational background, occupation, and alcohol and smoking status; these factors could also have an effect on TB treatment outcome.

### 3.7. Future Research Directions

Future studies should employ longitudinal designs to better understand how changes in viral load, CD4 count, smear status, and comorbidity status affect treatment outcomes over time. This will help us understand the relationships between each of these variables over the course of the treatment. Qualitative methods are needed to better understand why some patients fail to adhere to treatment and to identify barriers that lead to loss to follow-up. Future studies should explore the effectiveness of integrated care models, specifically if interventions can reduce the number of treatment failures, and improve viral load suppression. Further research is also needed to explore methods for improved management of comorbidities. Studies with larger and more diverse sample sizes should be used to allow for greater generalizability of the results. Research should explore the mechanisms behind the association between diabetes, hypertension, and TB treatment outcomes. Data collection systems should be improved to reliably capture information on sputum conversion.

## 4. Materials and Methods

This section outlines the methodological approach employed to investigate the determinants of Tuberculosis (TB) treatment outcomes in patients co-infected with HIV at selected level one hospitals in Lusaka, Zambia.

### 4.1. Study Design

A retrospective cohort study design was conducted by retrieving patient information from TB registers and smart care online systems.

### 4.2. Study Site

The study sites were Chipata, Chawama, and Chilenje level one hospitals in Lusaka. The three study sites were selected using a simple random selection method from the five level one hospitals in Lusaka. These three sites are in big urban communities in Lusaka with a high burden of HIV and TB. The Chilenje level one hospital has a catchment population of about 130,000 and consists of low-, medium-, and high-income level patients [35]. The Chipata level on hospital, despite having an equally high catchment population of about 133,000 and a high HIV/TB burden, mostly consists of low- and medium-income level patients [36]. The Chawama level one hospital, on the other hand, is located in one of the densely populated parts of Lusaka, serving about 129,000 patients and has higher rates of HIV and TB [17]. Therefore, this study captured data of TB/HIV co-infected patients of all income levels.

### 4.3. Study Population

The study population included all HIV-positive patients co-infected and diagnosed with Tuberculosis, accessed treatment between the year 2020 and 2022, were age 16 years and above, and had their treatment records at Chilenje, Chawama, and Chipata level one hospitals.

### 4.4. Sample Size and Calculation

The prevalence-based study technique was used to calculate the sample size. The sample size was calculated based on the prevalence of HIV among TB patients in Zambia. With the confidence interval of 95% and margin of error of 5%,

N = (Z^2^ P(1−P))/d^2^ (1)

where

Z = 0.95,
d = 0.05
P = 62% (Chanda-Kapata et al., 2017 [9])
N = 362

To address the design effect, the sample size was multiplied by 1.5 to give 543 as the overall sample size.

### 4.5. Sampling Method

The sample size was distributed among the three institutions using probability proportional to size. A systematic sampling method was employed to select the patient files. In this method, all eligible patient files were listed in a sequential order.

A random starting point was chosen, and then every third file on the list was picked for inclusion in the study sample.

### 4.6. Variables

The treatment outcome, as the dependent variable, was dichotomized into two categories: successful (including treatment completion and cure) and unsuccessful (including death, treatment failure, and loss to follow-up). The independent variables consisted of socio-demographic and clinical factors, such as age, sex, baseline BMI, baseline viral load, baseline CD4 count, HIV category, type of TB, TB treatment category, and the presence of other comorbidities.

### 4.7. Inclusion Criteria

All pulmonary TB/HIV co-infected patients who were treated for TB during the year 2020 to 2022 and were of age 16 years and above at the study sites

### 4.8. Exclusion Criteria

1. TB patients who are HIV-negative;
2. Participants with a confirmed mental disorder;
3. Trans-out patients (i.e., patients who are being transferred from the current care setting to another healthcare facility, unit, or level of care);
4. Trans-in patients (i.e., patients who are being transferred into the current care setting from another healthcare facility, unit, or level of care);
5. Patients with MDR TB.

Table 6 below presents the definitions of some of the key terms of this study.

### 4.9. Data Collection

Secondary clinical and demographic data of patients who met the eligibility criteria and were on TB treatment between January 2020 and December 2022 were abstracted from randomly selected patient files and TB registers from the three level one hospitals using a paper-based data collection tool. Demographic and clinical characteristics of the patients that were obtained from the TB registers and online smart care systems from the time the patients commenced treatment, at the end of the intensive phase (2 months), and finally, at the end of TB treatment, were categorized as follows: patient’s gender, recorded as male or female, age (in years), HIV category (newly diagnosed and already on ART), baseline CD4 count (dL/mL), baseline viral load (copies/mL), baseline weight and patient’s height, and other co-morbidities (yes or no). Dependent variables (treatment outcomes) were cure, death, treatment failure, treatment completed, and loss to follow-up. For patients who were TB smear-positive at the beginning of treatment, sputum conversion at follow-up visits was recorded from month 2 up to the end of TB treatment.

Data were entered into an excel sheet and were subjected to cleaning by removing any erroneous entries, duplicate data, and ambiguous parameters

### 4.10. Data Analysis

The statistical package for Social Sciences (SPSS) version 23 was used for data analysis. To describe categorical variables such as sex, category of HIV, type of TB, category of TB, and other comorbidities, frequency counts and percentages were used. Continuous variables such as age, baseline BMI, baseline CD4 count and the baseline viral load, were converted to categorical variables and analyzed using descriptive methods.

Data from 586 patient files were abstracted. Of these, 298 (50.8%) were from the Chilenje level one hospital, 163 (27.7%) from the Chipata level one hospital, and 126 (21.5%) from the Chawama level one hospital.

In the TB treatment program, when a patient is initiated upon treatment, the first two months are the intensive phase of treatment and involves a patient taking a combination of four anti-TB drugs under the directly observed therapy to enhance adherence. Upon completion of the intensive phase, for those who were sputum-positive at the beginning of treatment, the sputum samples were evaluated for conversion from positive to negative and followed up by sputum checks during subsequent visits.

### 4.11. Ethical Consideration

Ethical clearance was sought from the ERES converge IRB reference number 2024-May-007. Further approval was granted by NHRA reference number NHRAR-R-1659/07/06/2024. Permission to carry out the research was also sought from the Provincial Health Office, reference number LSKPHO/101/8/1 and from the Chilenje, Chawama, and Chipata level one hospitals. Requirement for patient consent was not considered because this study depended on secondary data from the patient’s files. Information that was collected from the patient’s files was confidential and only file numbers were indicated on the data collection sheet. The data collection sheets are kept safely under lock and key. Results for this research are sorely for academic purpose and will be released to the designated authorities and through publications.

## 5. Conclusions

This study has illuminated several key determinants of TB treatment outcomes among TB/HIV co-infected patients in Lusaka, Zambia. It highlights the need for an integrated, patient-centered approach to TB/HIV care, with a focus on viral suppression, tailored care for smear-positive TB, management of comorbidities, and a focus on monitoring early treatment response. By implementing the recommendations outlined here, healthcare systems can take a step forward in improving TB treatment success and reducing mortality for this vulnerable population. Continued research is needed to refine treatment strategies and address the remaining challenges in TB/HIV co-infection management.

## Figures and Tables

**Table 1:** Demographic and baseline clinical characteristics of study participants [17].

Variable	Description	Frequency	Percent
Hospital	Chawama	125	21.3
	Chilenje	298	50.9
	Chipata	163	27.8
Age	Under 18	1	0.2
	18 to 24	57	9.8
	25 to 44	361	61.6
	45 to 64	139	23.8
	65 and above	26	4.5
Sex	Male	331	56.6
	Female	254	43.4
Baseline BMI	Under 18.5	151	25.8
	18.5 to 24.9	289	49.3
	25 to 29.9	95	16.2
	30 and above	43	7.3
Baseline Viral Load	Under 1000	25	4.3
	1000 to 9999	95	16
	10,000 to 99,000	255	43.5
	100,000 to 199,999	129	22
	200,000 and above	83	14.2
Baseline CD4	Under 200	185	31.6
	200 to 349	238	40.6
	350 to 500	101	17.4
	Over 500	58	10
Category of HIV	Newly diagnosed	252	44
	On ART	328	56
Type of TB	Smear-Positive	124	21.2
	Smear-Negative	461	78.8
Category of TB	New case	389	66.5
	Retreatment	196	33.5
Other Comorbidities	Yes	96	16.6
	No	487	83.4
Comorbidity Type	Hypertension	51	53.1
	Diabetes Mellitus	24	25
	Hypertension/Diabetes	21	21.9
Intensive Phase Duration	Up to 2 months	527	98.3
	Over 2 months	9	1.7
Smear Conversion	Positive	2	0.3
	Negative	527	90.1
	Unknown/Not done/Missing	56	9.6

**Table 2:** Treatment Outcomes of Study Participants.

Variable	Description	Frequency	Percent
At End of Intensive Phase	Died	40	6.9
	Alive	541	93.1
At End of Continuous Phase (6 months)	Cured	69	11.8
	Treatment Completed	405	69.5
	Loss to follow-up	36	6.2
	Died	73	12.5

**Table 3:** Chi-square analysis of associations with treatment outcome at 6 months.

Variable	Pearson Chi-Square	Df	*n* of Valid Cases	*p*-Value
Age	16.333	12	581	0.176
Sex	1.683	3	582	0.641
Baseline BMI	10.095	9	575	0.343
Baseline Viral Load	39.080	12	583	0.001
Baseline CD4	19.807	9	579	0.019
Category of HIV	14.371	3	577	0.002
Type of TB	291.250	3	582	0.001
Category of TB	5.954	3	582	0.114
Other Comorbidities	4.742	6	581	0.577
Comorbidity Type	14.222	6	582	0.027
Intensive phase duration	6.249	3	534	0.100
Smear Conversion At Follow-Up	64.824	6	583	0.001

**Table 4:** Hierarchical logistic regression analysis of predictors of TB treatment outcomes.

Variable	Model 1		Model 2		Model 3		Model 4		Model 5		Model 6	
	Sig.	Exp(B)	Sig.	Exp(B)	Sig.	Exp(B)	Sig.	Exp(B)	Sig.	Exp(B)	Sig.	Exp(B)
BV	0.001		0.004		0.004		0.003		0.004		0.135	
BV (1)	0.069	0.299	0.118	0.237	0.114	0.229	0.068	0.181	0.085	0.193	0.483	0.496
BV (2)	0.004	0.344	0.028	0.263	0.027	0.256	0.017	0.227	0.017	0.216	0.133	0.358
BV (3)	0.000	0.297	0.000	0.198	0.000	0.193	0.000	0.179	0.000	0.181	0.016	0.319
BV (4)	0.035	0.511	0.019	0.461	0.018	0.458	0.022	0.465	0.025	0.464	0.497	0.775
BCD4			0.443		0.458		0.425		0.368		0.463	
BCD4 (1)			0.675	0.741	0.714	0.766	0.608	0.687	0.478	0.588	0.707	0.742
BCD4 (2)			0.696	1.285	0.674	1.313	0.762	1.218	0.882	1.104	0.679	1.340
BCD4 (3)			0.963	1.026	0.959	1.029	0.942	0.961	0.858	0.905	0.682	1.276
CHIV (1)					0.794	0.930	0.732	0.909	0.902	0.966	0.708	1.117
TTB (1)							0.002	0.345	0.001	0.326	0.001	0.277
CT									0.028		0.038	
CT (1)									0.068	0.363	0.039	0.300
CT (2)									0.064	2.506	0.169	2.058
SCo											0.000	
SCo (1)											1.000	6.75 × 10^8^
SCo (2)											0.000	0.137
Omnibus Tests of Model Coefficients												
Chi-square		17.753		2.683		0.069		11.673		7.879		35.24
Sig.		0.001		0.443		0.793		0.001		0.019		0
Model Summary												
−2 Log likelihood		530.63		527.94		527.8		516.20		508.33		473.09
Cox and Snell R Square		0.031		0.035		0.035		0.055		0.068		0.123
Nagelkerke R Square		0.050		0.057		0.057		0.089		0.110		0.200
Hosmer and Lemeshow Test												
Chi-square		0.000		1.491		2.349		6.517		1.802		5.939
Sig.		1.000		0.960		0.885		0.589		0.970		0.654

**Table 5:** Summary of hierarchical logistic regression analysis.

Variable	Code	Categories	Odds Ratio EXP(B)	*p*-Value
Baseline viral load	BV	Under 1000		0.135
	BV (1)	1000 to 9999	0.496	0.483
	BV (2)	10,000 to 99,999	0.358	0.133
	BV (3)	100,000 to 199,999	0.319	0.016
	BV (4)	200,000 and above	0.775	0.497
Baseline CD4	BCD4	Under 200		0.463
	BCD4 (1)	200–349	0.742	0.707
	BCD4 (2)	350–500	1.340	0.679
	BCD4 (3)	Over 500	1.276	0.682
Category of HIV	CCHIV (1)		1.117	0.708
Type of TB	TTB (1)		0.277	0.001
Comorbidity type	CT	Hypertension		0.038
	CT (1)	Diabetes Mellitus	0.300	0.039
	CT (2)	Hypertension/Diabetes	2.058	0.169
Smear conversion	SCo	positive		0.001
	SCo (1)	negative	6.75 × 10^8^	1.00
	SCo (2)	Unknown/missing/not done	0.137	0.001

**Table 6:** Definition of treatment outcome according to the Ministry of Health National TB and Leprosy Program-consolidated guidelines [17].

Term	Definition
Cured	A pulmonary TB patient with bacteriologically confirmed TB at the beginning of treatment who was smear- or culture-negative in the last month of treatment and on at least one previous occasion.
Completed	A TB patient who completed treatment without evidence of failure BUT with no record to show that sputum smear or culture results in the last month of treatment and on at least one previous occasion were negative, either because tests were not performed or because results are unavailable.
Treatment failed	A TB patient whose sputum smear or culture is positive at month 5 or later during treatment.
Not evaluated	A TB patient for whom no treatment outcome is assigned. This includes cases ‘transferred out’ to another treatment unit as well as cases for whom the treatment outcome is unknown to the reporting unit.
Lost to follow-up	A TB patient who did not start treatment or whose treatment was interrupted for 2 consecutive months or more.
Died	A TB patient who dies for any reason before starting or during treatment.
Treatment success	The sum of the cases that were cured and completed treatment.
Treatment failure	Sum of the cases that were regarded as treatment failed, died, lost to follow-up, and not evaluated.

## Data Availability

The original contributions presented in this study are included in the article. Further inquiries can be directed to the corresponding author(s).
